# Effect of Thermophilic Nitrate Reduction on Sulfide Production in High Temperature Oil Reservoir Samples

**DOI:** 10.3389/fmicb.2017.01573

**Published:** 2017-08-29

**Authors:** Gloria N. Okpala, Chuan Chen, Tekle Fida, Gerrit Voordouw

**Affiliations:** ^1^Petroleum Microbiology Research Group, Department of Biological Sciences, University of Calgary, Calgary AB, Canada; ^2^State Key Laboratory of Urban Water Resource and Environment, Harbin Institute of Technology Harbin, China

**Keywords:** oil field, seawater, sulfate reduction, nitrate reduction, microbial community

## Abstract

Oil fields can experience souring, the reduction of sulfate to sulfide by sulfate-reducing microorganisms. At the Terra Nova oil field near Canada’s east coast, with a reservoir temperature of 95°C, souring was indicated by increased hydrogen sulfide in produced waters (PW). Microbial community analysis by 16S rRNA gene sequencing showed the hyperthermophilic sulfate-reducing archaeon *Archaeoglobus* in Terra Nova PWs. Growth enrichments in sulfate-containing media at 55–70°C with lactate or volatile fatty acids yielded the thermophilic sulfate-reducing bacterium (SRB) *Desulfotomaculum*. Enrichments at 30–45°C in nitrate-containing media indicated the presence of mesophilic nitrate-reducing bacteria (NRB), which reduce nitrate without accumulation of nitrite, likely to N_2_. Thermophilic NRB (tNRB) of the genera *Marinobacter* and *Geobacillus* were detected and isolated at 30–50°C and 40–65°C, respectively, and only reduced nitrate to nitrite. Added nitrite strongly inhibited the isolated thermophilic SRB (tSRB) and tNRB and SRB could not be maintained in co-culture. Inhibition of tSRB by nitrate in batch and continuous cultures required inoculation with tNRB. The results suggest that nitrate injected into Terra Nova is reduced to N_2_ at temperatures up to 45°C but to nitrite only in zones from 45 to 65°C. Since the hotter zones of the reservoir (65–80°C) are inhabited by thermophilic and hyperthermophilic sulfate reducers, souring at these temperatures might be prevented by nitrite production if nitrate-reducing zones of the system could be maintained at 45–65°C.

## Introduction

Oil reservoir souring, the reduction of sulfate to sulfide by sulfate-reducing microorganisms (SRM), and its control with nitrate has been studied extensively in shallow, low temperature reservoirs, which support the growth of mesophilic microbes throughout. Injection of water with 1 mM sulfate, amended with 2 mM nitrate, caused the emergence of sequential zones of nitrate-reduction, sulfate-reduction and methanogenesis along the water flow path. Although microbial communities in produced waters (PW) were dominated by methanogens, SRB and NRB were present and were readily activated when samples were grown under nitrate- or sulfate-reducing conditions. All three of these activities were, therefore, easily established in bioreactors or microcosms containing nitrate, sulfate and excess volatile fatty acids irrespective whether the inoculum was a field sample or a derived enrichment (Lambo et al., 2008; Voordouw et al., 2009; Callbeck et al., 2011; Chen et al., 2017). In contrast studying microbial communities derived from deep, high temperature reservoirs is much more complex. Because the zones of microbial activities are superimposed on a steep gradient of increasing temperature in the near injection wellbore region (NIWR).

**FIGURE 1:**
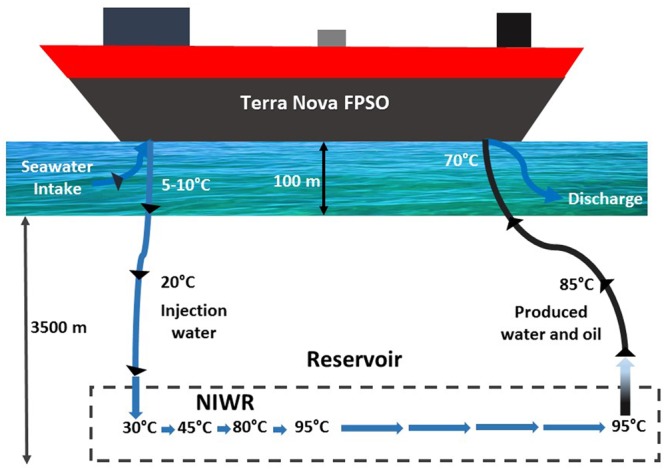
Schematic of seawater injection from the Terra Nova Floating Production Storage and Offloading (FPSO) vessel, as explained in the text. The near injection wellbore region (NIWR) has mesophilic (30–45°C), thermophilic (45–80°C) and abiotic (80–95°C) zones.

For instance, in the Terra Nova field, located 350 km from the east coast of Newfoundland, oil is produced from a depth of 3200–3700 m below the sea floor, where the reservoir temperature is 95°C (Haugen et al., 2007). Due to the harsh operating conditions existing in this region, the oilfield is operated through a Floating Production Storage and Offloading (FPSO) vessel from where flexible manifold pipes are connected to the subsea system (Howell et al., 2001). Following its intake, cold seawater is warmed during its downward travel, reaching 30°C upon injection in the reservoir (**Figure 1**). This temperature further increases in the NIWR, in which the temperature changes from that of the injected water to that of the bulk of the reservoir (Eden et al., 1993). The NIWR thus consists of a succession of mesophilic (30–45°C), thermophilic (45–80°C) and abiotic (80–95°C) zones, assuming that microbial life in the reservoir does not extend beyond 80°C (Magot, 2005; Liebensteiner et al., 2014; Fida et al., 2016). Following travel through the abiotic bulk of the reservoir (95°C), the temperature of the PW and oil mixture will cool to 70°C, when it travels upward to the FSPO, allowing renewed growth of thermophilic but not of mesophilic microorganisms. Produced oil and water are then separated in the FPSO with cleaned but still hot PW being discharged into the ocean (**Figure 1**).

A variety of mesophilic and thermophilic NRB (tNRB), SRM, and fermentative bacteria, as well as methanogenic *Archaea* have been obtained from or detected in injected seawater and PW from high temperature reservoirs (Beeder et al., 1995; Nilsen et al., 1996a,b; Slobodkin et al., 1999; Orphan et al., 2003; Nazina et al., 2006; Gittel et al., 2009, 2012; Zhang F. et al., 2012; Aüllo et al., 2013; Lenchi et al., 2013; Agrawal et al., 2014). Their positioning in the NIWR depends on the temperature dependence of their activity. However, data on this for multiple isolates from the same field are lacking. Study of the physiology of pure cultures or enrichments has indicated that tNRB reduce nitrate to nitrite, but do not reduce nitrite, e.g., to di-nitrogen (N_2_) (Fida et al., 2016). Hence, addition of nitrate to a culture of thermophilic SRB (tSRB) in which tNRB are present would strongly inhibit tSRB activity, because nitrite is such a strong and specific SRB inhibitor (Greene et al., 2003; Haveman et al., 2004). At low temperature mesophilic NRB can persist in cultures of mesophilic SRB in the absence of nitrate by switching to fermentative metabolism. However, coculturing of tNRB and SRB is more difficult. For example sulfide production by two tSRM enrichments from North Sea fields at 60°C, harboring *Thermodesulforabdus* or *Archaeoglobus*, was not inhibited by 10 mM nitrate, indicating absence of tNRB (Kaster et al., 2007). Both of these enrichments were strongly inhibited by only 0.25 mM nitrite. Likewise, Reinsel et al. (1996), demonstrated that injecting as little as 0.71 mM nitrate inhibited sulfate reduction in souring bioreactors at 60°C due to its reduction to nitrite. However, this inhibition could be lost in bioreactors, which had been injected with sulfate only for a prolonged period of time, requiring re-inoculation with tNRB.

Our objectives in researching the Terra Nova system were to determine the community composition, as well as the temperature dependence of reduction of nitrate to nitrite, of nitrite to nitrogen and of sulfate to sulfide for samples of injection and PWs, of derived enrichments and of pure cultures. This may allow mapping of the location of taxa and their activities in the temperature versus distance profile of the NIWR. Further study of cocultures of tSRB and NRB was also pursued to improve understanding of souring control in systems with a temperature gradient as in the NIWR.

## Materials and Methods

### Sample Collection and Physicochemical Analysis

Samples were collected on-board the FPSO and were sent in sealed containers. Three sets of injection water (IW) and PW samples were received from Terra Nova oil fields in January 2014, January 2015 and May 2015. The samples were either shipped in 3 L airtight metal canisters or in 1 L Nalgene bottles, with 2 or 3 L of each sample provided (**Table 1**). The sample bottles or canisters were filled to the brim and sealed tightly to exclude air. Upon arrival, the samples were stored at room temperature in an anoxic chamber with an atmosphere of 10% CO_2_ and 90% N_2_ (N_2_-CO_2_; Praxair, Calgary, AB, Canada). An aliquot of 200 ml of each sample was centrifuged for 15 min at 11,200 × *g* to pellet biomass, the pellets were frozen at -20°C for use in DNA extraction. The salinity as molar equivalent (Meq) of NaCl was determined from the conductivity measured with an Orion conductivity cell (model 013005MD). Sulfide and ammonium concentrations in the water samples were determined spectrophotometrically using the methylene blue method (Cline, 1969) and the indophenol method (Cornish Shartau et al., 2010), respectively.

**Table 1:** Physicochemical analyses of injection water (IW) and produced water (PW) samples.

Water	pH	NaCl	Sulfate	Acetate	Propionate	NH_4_^+^
chemistry		(Meq)	(mM)	(mM)	(mM)	(mM)
**Injection water (IW) 2014-2015**						
IW1\_14	6.74	0.48	28	0.11	0	0.11
IW1\_15	6.51	0.55	27.6	0.02	0	0.34
IW5\_15	6.42	0.53	19.8	0	0	0.32
Average ± SD	6.56 ± 0.17	0.52 ± 0.04	25.1 ± 4.6	0.04 ± 0.06	0	0.26 ± 0.13
**Produced Water (PW) 2014-2015**						
PW1\_14	6.75	0.4	16.8	2.26	0.24	1.24
PW1\_15	6.97	0.69	22.14	1.85	0.08	1.4
PWC2\_5\_15	6.7	0.55	13.8	0.28	0.02	0.46
PWF2\_5\_15	6.7	0.64	8.9	1.76	0.17	1.06
Average ± SD	6.78 ± 0.13	0.57 ± 0.13	15.4 ± 5.5	1.54 ± 0.87	0.13 ± 0.10	1.04 ± 0.41

Nitrate and nitrite concentrations were measured by high performance liquid chromatography (HPLC), using a Waters 600E HPLC (Waters Corp, Milford, MA, United States) which was fitted with a Waters 2489 UV/Visible detector, set at 200 nm and an IC-PAK^TM^ anion column HC (150 × 4.6 mm, waters), and eluted with a sodium borate-gluconate (2%) buffer containing 12% acetonitrile and 2% butanol. Sulfate, was measured with the same column using a Waters 432 conductivity detector at a flowrate of 2 ml/min. Samples for anion assay were prepared by centrifugation at 14,000 rpm for 5 min, after which 100 μl of supernatant was added to 400 μl of the prepared buffer solution in a vial. Volatile fatty acid (VFA) concentrations of field samples were analyzed with a Waters 2487 UV detector at 210 nm, with a Prevail organic acid (OA) 5u column (250 × 4.6 mm, Alltech, Guelph, ON, Canada) at a flow rate of 1.0 ml/min. Field samples (1 ml) were centrifuged and 300 μl of the supernatant was acidified in a vial with 20 μl of 1M H_3_PO_4_ before elution with 25 mM KH_2_PO_4_ (pH 2.5).

### Microbial Enumeration of SRB and Acid-Producing Bacteria (APB)

Most probable number (MPN) determinations were done using 48-well cell culture plates (Shen and Voordouw, 2015) for enumerating SRB and acid-producing bacteria (APB) using media containing lactate and sulfate or glucose and phenol red, respectively. Plates were incubated at 30°C or 60°C inside an anaerobic jar for 1 month. MPNs were calculated by comparing the pattern of positive wells to a probability table for MPN tests done using triplicate series of dilutions.

### Activity of SRB and NRB

Microbial activity tests were done by inoculating 10% (v/v) of sample in modified Coleville synthetic brine (CSB) medium A with 0.5 M NaCl. CSBA medium had the following composition (g/L): NaCl, 29.3; CaCl_2_.2H_2_O, 0.15; MgCl_2_.5H_2_O, 0.4; NH_4_Cl, 0.25; KCl, 0.5; KH_2_PO_4_, 0.2; resazurin (1%), 2–3 drops. After autoclaving, trace elements, 1 ml; selenate-tungstate, 1 ml; 1 M NaHCO_3_, 30 ml were added and the pH was adjusted to 7.4–7.6 using 1 M HCl (Hubert et al., 2003). Fifty ml of the CSBA medium was added to 122 ml serum bottles, which were sealed with a butyl rubber stopper, crimped with an aluminum cap and flushed with N_2_-CO_2_ gas (90–10%) for 5 min to exclude oxygen. Sulfate, nitrate, VFA, lactate and sulfide were added to these media in final concentrations as indicated. The media in the serum bottles were inoculated with 10% (v/v) of IW or PW samples. Following inoculation, the serum bottles were incubated at 30 or 60°C. Aliquots of 0.5 ml were taken at different time intervals to determine the concentrations of sulfide, sulfate, nitrite and nitrate. Sulfide concentrations were determined immediately after each sampling, and the remainders of the samples were frozen (-20°C) for further analysis of sulfate, nitrate and nitrite by HPLC.

### Enrichment of Thermophilic SRB and NRB Consortia from Field Samples

To increase the probability of cultivating tSRB and NRB from the water samples, the biomass in the water samples was concentrated by either filtration or centrifugation. For filtration, 250 mL of sample were filtered through a 0.2 μm filter, after which the biomass concentrated on the surface of the filter, was inoculated into 30 mL of the filtrate or of CSBA. For centrifugation, 250 ml of PW or IW were centrifuged at 11,200 × *g* for 15 min. After centrifugation, the supernatant was poured off, and the pellets formed were re-suspended with 5 ml of the supernatant.

Aliquots (20 ml) of CSBA medium were dispensed into 50 ml serum bottles, and sealed with rubber stoppers and aluminum crimps. The medium was flushed with N_2_-CO_2_. To the SRB media, 20 mM lactate and 10 mM sulfate or 6 mM VFA and 10 mM sulfate were added, while to the NRB media, 20 mM lactate and 10 mM nitrate were added. The inoculated media were incubated at 60°C. Samples were taken periodically with an N_2_-CO_2_ flushed syringe. The nitrate, sulfate and nitrite concentrations were determined using HPLC, while sulfide was measured colorimetrically.

### Temperature Dependence of Sulfate and Nitrate Reduction

IW and PW samples collected in 2015 were inoculated into CSBA medium containing 3 mM VFA (3 mM each of acetate, propionate and butyrate) and 10 mM nitrate or 20 mM lactate and 10 mM sulfate. Following inoculation, the incubations were done at 30, 40, 45, 50, 55, 60, 65, and 70°C. Aliquots of 0.5 ml were withdrawn periodically from the incubations to monitor sulfide, sulfate, nitrate and nitrite concentrations.

### Isolation and Identification of tNRB Strains

Thermophilic NRB enrichments derived from IW1_14 grown at 60°C or derived from IW5_15 grown at 50°C were 10-fold serially diluted in CSBA medium and 100 μl of the dilutions was plated on a 2% CSBA-agar medium containing 3 mM VFA and 10 mM nitrate. The plates were incubated at 50°C or 60°C in anaerobic jars flushed with N_2_-CO_2_. Individual colonies were picked and grown in CSBA medium with 3 mM VFA and 10 mM nitrate. To identify the isolates, DNA was extracted and 16S rRNA gene amplicons were obtained using primers 27F and 1525R. Sanger sequencing of the amplicons was done at the Core DNA Services Laboratory of the University of Calgary.

### Effect of Nitrate and Nitrite on Sulfate Reduction by tSRB

To assess the inhibition of sulfide production in tSRB consortia using nitrate or nitrite, 10% (v/v) of tSRB enrichment was grown in CSBA medium containing 20 mM lactate and 10 mM sulfate. Also, a tNRB mixed culture of *Geobacillus* sp. strain TK004 and TK005 was prepared by inoculating glycerol stocks of each strain into CSBA medium with 0.25 M NaCl, containing 20 mM lactate and 10 mM nitrate. The effectiveness of tNRB activity in inhibiting sulfate reduction was monitored by adding tNRB and nitrate at the start (0 h) or in mid-log phase of a tSRB culture.

The effect of nitrate and nitrite on sulfate reduction by tSRB growing in continuous culture was also assessed. A continuous culture of tSRB was started by inoculating 10% of a 48-h tSRB culture into 90 mL CSBA containing 10 mM lactate and 5 mM sulfate. Once all sulfate was reduced, a multichannel peristaltic pump was used to pump the same medium at a flow rate of 33 ml/d (dilution rate 0.33 d^-1^). To test the effect of nitrate addition on sulfate reduction, CSBA medium containing both 5 mM nitrate and 5 mM sulfate was injected into the tSRB culture in medium with sulfate only. To evaluate the effect of tNRB addition on sulfate reduction, tNRB were grown in CSBA medium containing 5 mM nitrate and 10 mM lactate. The cells were harvested at mid-log phase and washed with CSBA to remove any residual nitrate or nitrite. The cell pellets were re-suspended in CSBA medium and then inoculated into the tSRB culture. The effect of nitrite on sulfate reduction by tSRB was monitored by adding 0.125, 0.25, or 1 mM nitrite to the injection medium.

### Microbial Community Analysis

DNA was isolated from 200 ml of PW and IW samples using the Fast DNA Spin Kit for Soil and the FastPrep Instrument (MP Biomedicals, Santa Ana, CA, United States) as per the manufacturer’s instructions. The extracted DNA was quantified using a Qubit fluorimeter (Invitrogen). Pyrosequencing of 16S amplicons was done for 2014 samples, whereas Illumina Miseq sequencing was done for 2015 samples.

For pyrosequencing PCR amplification was for 25 cycles with 16S primers 926Fw and 1392R, followed by 10 cycles with FLX titanium primers 454T_RA_X and 454T_FwB, as described in An et al. (2013). Purified 16S amplicons (20 ng each) were sequenced at the Genome Quebec and McGill University Innovation Centre, Montreal, Quebec with a Genome Sequencer FLX Instrument, using a GS FLX Titanium Series Kit XLR70 (Roche Diagnostics Corporation).

For Illumina Miseq sequencing 16S rRNA genes of the extracted DNA were amplified using a two-step PCR procedure with each reaction of 50 μl volume containing premade reagents mixed in proportion as per manufacturer’s instructions (Thermo- Scientific). The first PCR used 16S primers 926Fi5 and 1392Ri7, as indicated elsewhere (Menon and Voordouw, 2016). The PCR product obtained was purified and quantified and was then used for the second PCR reaction, which used primer P5-S50X-OHAF and P7-N7XX-OHAR for 10 cycles, as described elsewhere (Menon and Voordouw, 2016). The resulting purified PCR product was sequenced using the 300PE (paired-end) MiSeq protocol on an Illumina Miseq system at the Department of Geosciences, University of Calgary. The 300PE reads were merged using PEAR 0.9.6 with a 50 bp overlap and were further processed with a 420 bp cutoff of amplicon size using MetaAmp, a 16S rRNA data analysis pipeline, developed by the Energy Bioengineering Group, Department of Geosciences, University of Calgary. MetaAmp was also used for bioinformatic analysis^1^.

All sequences have been submitted to NCBI Sequence Read Archive (SRA) under Bioproject accession number PRJNA181037, with biosample number SAMN06645415 and SAMN06645441.

## Results

### Physicochemical Analyses and Most Probable Numbers

The average salinity of Terra Nova IWs was 0.52 ± 0.04 Meq of NaCl, which was similar to that of produced waters (**Table 1**: 0.57 ± 0.13 Meq of NaCl). The concentrations of sulfate in IW samples (25.1 ± 4.6 mM) were higher than those of produced waters, (15.4 ± 5.5 mM). PW samples had higher concentrations of acetate, propionate and ammonium than IW samples (**Table 1**). All samples had a near neutral pH. Concentrations of nitrate, nitrite and sulfide were zero for all the samples.

The MPNs for SRB, determined by incubation at 30 or 60°C, were below the detection limit in both the IW and PW samples. IW samples had some mesophilic APB (3.6/ml; 30°C), but no thermophilic APB (60°C). No mesophilic or thermophilic APB were detected in the PW samples. Overall these results indicate that only small numbers of bacteria, culturable on the media used, were present in the samples.

### Microbial Community Analysis of IW1_14 and PW1_14

Results derived from pyrosequencing of 16S rRNA amplicons for the 2014 samples and from Illumina sequencing of 16S rRNA amplicons for the 2015 samples are presented in Supplementary Tables S1, S2, respectively. The microbial community in PW1_14 had significant fractions of potentially thermophilic *Euryarchaeota* (Supplementary Table S1), including *Methanothermococcus* (7.1%), *Methermicoccus* (2.2%) *Thermococcus* (2.2%) and *Archaeoglobu*s (1.7%). The majority of *Bacteria* in the PW1_14 community were thermophiles belonging to the genera *Thermoanaerobacter* (78.2%) and *Thermosipho* (0.7%). Members of the *Deltaproteobacteria* included the tSRB *Desulfonauticus* (0.3%; Piceno et al., 2014). The microbial community in injection water IW1_14 consisted mainly of aerobic mesophilic marine bacteria. About 33.2% of the total reads were affiliated with the genus *Neptuniibacter*, which reduces nitrate to nitrite at temperatures between 4 and 33°C (Arahal et al., 2007; Gutierrez et al., 2013). Other potential hydrocarbon degraders were *Thalassospira* (5.7%), *Alcanivorax* (4.5%) and *Cycloclasticus* (3.0%) (Gutierrez et al., 2013). *Alcanivorax* spp. are moderately halophilic alkane degraders which reduce nitrate to nitrite and N_2_ (Nakano et al., 2009; Mayumi et al., 2011; McGenity et al., 2012; Singh et al., 2014). The nitrate-reducing *Marinobacter* was present at 0.5% (Supplementary Table S2) (Li et al., 2013; Stepanov et al., 2016). Contrary to the community in PW1_14, that of IW1_14, harbored no thermophiles (Supplementary Table S1: entries #12, 26-31).

Few potentially thermophilic taxa were found in samples collected in 2015. These were dominated by *Alpha-* and *Gammaproteobacteria*, but lacked *Euryarchaeo*ta. IW5_15 had a high fraction of the sulfur-oxidizing *Thiomicrospira* (Table S2, entry #21, 42%), which was not found in the other samples. Most of the dominating taxa are considered mesophilic. The community in PW1_15 had small fractions of *Methanothermococcus* and *Thermococcus* (Supplementary Table S2: entries #30 and 31).

### Thermophilic Enrichments of Field Samples

**FIGURE 2:**
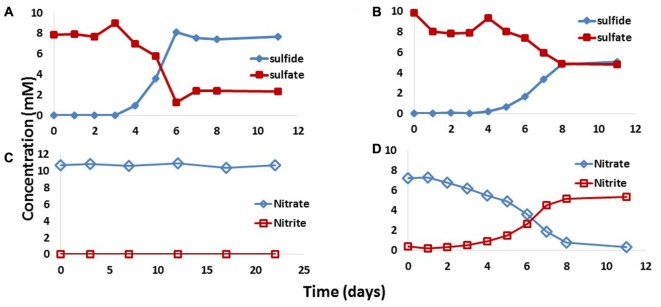
Activity of tSRB and tNRB observed with 50-fold concentrated inocula of Terra Nova produced water PW1\_14 and injection water IW1\_14. Incubations were with PW1\_14 with 10 mM sulfate and 20 mM lactate **(A)**, PW1\_14 with 10 mM sulfate and 3 mM VFA **(B)**, PW1\_14 with 10 mM nitrate and 20 mM lactate **(C)** and IW1\_14 with 10 mM nitrate and 20 mM lactate **(D)**. Data are averages of duplicate incubations.

Because Terra Nova samples had few culturable bacteria, as judged by MPN assays, enrichment of tSRB and tNRB consortia was done with concentrated inocula. Injecting concentrated PW1_14 and IW1_14 in media gave the results indicated in **Figure 2**. Activity of tSRB was detected in lactate-sulfate medium after 3–6 days of incubation at 60°C (**Figure 2A**), whereas tSRB activity was detected in VFA-sulfate medium after 4 to 8 days of incubation (**Figure 2B**). No tSRB activity was detected in medium inoculated with concentrated IW1_14 (results not shown). tNRB activity was observed in medium with 20 mM lactate and 10 mM nitrate inoculated with concentrated IW1_14 (**Figure 2D**). Nitrate was reduced to nitrite, which was not reduced further. No tNRB activity was observed with concentrated PW1_14 (**Figure 2C**).

Nitrate-reducing enrichments using 50-fold concentrated inocula of samples PW1_15 and IW1_15 were done at different temperatures (Supplementary Figure S1). Nitrate was completely reduced within 24 h at 30 and 40°C (Supplementary Figures S1A–D). Nitrite appeared transiently up to 4.8 mM in the incubation with IW1_15 at 40°C (Supplementary Figure S1B), whereas 1.0 mM nitrite persisted in the incubation with PW1_15 at 40°C (Supplementary Figure S1E). At 45°C nitrate was slowly reduced to nitrite in the medium inoculated with concentrated IW1_15 PW (Supplementary Figure S1C). Reduction of 3 mM nitrate was observed with PW1_15 without production of nitrite (Supplementary Figure S1F). Incubations at 50 to 70°C showed no tNRB activity (results not shown). SRB activity was observed for concentrated PW1_15 in medium with VFA and sulfate at 60°C (results not shown).

**FIGURE 3:**
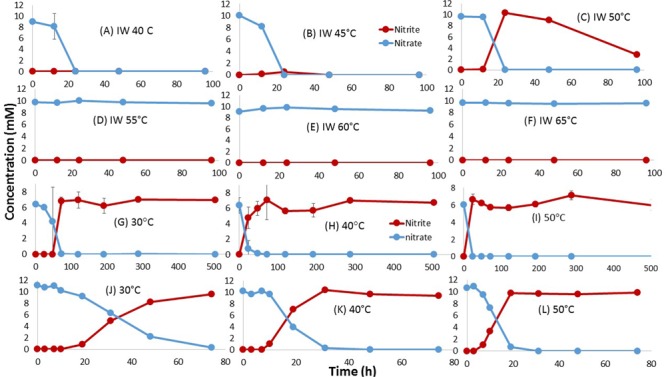
Effect of incubation temperature on nitrate reduction in cultures derived from IW5\_15. Data are for primary enrichments **(A–F)**, for secondary enrichments inoculated with primary enrichment **(C)** grown at 50°C **(G–I)** and for a pure culture isolate, identified as *Marinobacter* sp., obtained at 50°C **(J–L)**. No growth was observed for the cultures in **(G–L)** at 55°C or higher temperature. The CSBA medium contained 3 mM VFA and 10 mM nitrate.

Use of 50-fold concentrated inocula of IW5_15 indicated rapid reduction of nitrate at 40, 45, and 50°C (**Figures 3A–C**). Nitrite was not detected at 40 and 45°C, but persisted in the 50°C incubation (**Figure 3C**). No NRB activity was found at 55, 60, and 65°C (**Figures 3D–F**). No SRB activity was found at any of these temperatures. No NRB or SRB activity was found with concentrated inocula of PW5_15 at 40–65°C.

Thus, significant tSRB and tNRB activity at 60°C were observed in the produced water and IW samples collected in January of 2014, respectively. Samples collected in January and May 2015 gave tNRB activity at lower temperature (50°C). These results are in agreement with the increased presence of thermophiles in the 2014 samples, as compared to the 2015 samples, indicated by microbial community analyses (Supplementary Tables S1, S2).

### NRB Enrichment at 50°C and 60°C and Isolation of tNRB

The tNRB enrichment obtained at 50°C (**Figure 3C**) was further evaluated for effect of temperature on nitrate reduction, growth and community composition. The results in **Figures 3G–I** showed that in incubations at 30, 40, and 50°C, nitrate was reduced to nitrite and no further. Nitrate was completely reduced at 30°C within 72 h (**Figure 3G**), at 40°C within 48 h (**Figure 3H**) and at 50°C within 24 h (**Figure 3I**). Nitrite accumulated at all three temperatures. No nitrate reduction was observed above 50°C. High cell density of these cultures was observed at 30, 40, and 50°C, but not at higher temperatures (Supplementary Figure S2A), indicating the nitrate reducers to be facultative tNRB. Mesophilic NRB, which reduced nitrite (**Figures 3A,B**) were no longer present in this enrichment. Microbial community data for the incubations in **Figures 3G–I** indicated *Marinobacter* spp. as the dominant NRB present at 99.6%, 68 and 99.7% respectively (Supplementary Figure S2B). A pure culture isolate, obtained from the enrichment in **Figure 3I** and grown at 50°C, was identified as *Marinobacter* sp. GN001 (KY818661). Growth of this isolate at temperatures ranging from 30 to 60°C, indicated that it reduced nitrate, but not nitrite, at 30, 40, and 50°C (**Figures 3J–L**). Nitrate was not reduced at 60°C (results not shown). Nitrate reduction proceeded most rapidly at 40 and 50°C and more slowly at 30°C, like the enrichment in **Figures 3G–I**.

**FIGURE 4:**
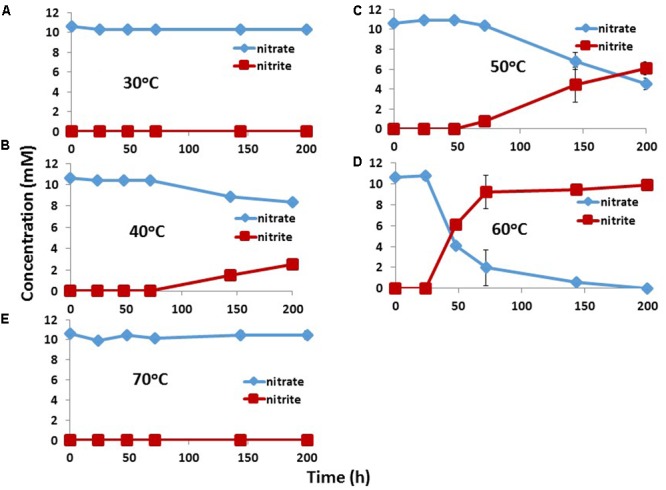
Effect of incubation temperature on nitrate reduction by a pure culture isolate *Geobacillus* sp. TK004, obtained from IW1\_14. Data are averages for duplicate incubations; standard deviations are shown. The medium contained 3 mM VFA and 10 mM nitrate. 30°C **(A)**, 40°C **(B)**, 50°C **(C)**, 60°C **(D)**, and 70°C **(E)**.

Pure culture tNRB isolates TK004 and TK005 were obtained from IW1_14 at 60°C. Both were identified as *Geobacillus* spp. by 16S rRNA sequencing. Nitrate reduction by TK004 as a function of temperature indicated maximal activity at 60°C, lower activity at 50 and 40°C and no activity at 30 and 70°C. Nitrate was reduced to nitrite only (**Figure 4**).

### Enrichment of tSRB

**FIGURE 5:**
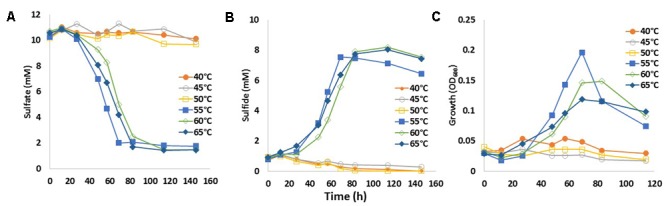
Effect of incubation temperature on growth and sulfate reduction by tSRB consortia enriched from PW1\_14. The concentration of sulfate **(A)** and of sulfide **(B)**, as well as the cell density as OD_600_ **(C)** are presented as a function of time. Data are averages for duplicate incubations. The medium contained 20 mM lactate and 10 mM sulfate.

The temperature dependence of the rate of sulfate reduction to sulfide was determined for a tSRB consortium previously enriched from PW1_14 at 60°C (**Figure 2A**). Reduction of sulfate to sulfide was observed at 55, 60, and 65°C (**Figures 5A,B**), but not at lower temperatures. Significant increases in biomass were also only observed at 55, 60, and 65°C (**Figure 5C**). Reduction of sulfate to sulfide was observed at 70°C after 300 h at a very slow rate (Supplementary Figure S3). This finding was further supported by the community data from 55 to 65°C incubations, when compared to the inocula used for the experiment (Supplementary Table S3). Bioinformatic analysis of quality controlled Illumina reads indicated that the tSRB consortium was more diverse than the 55, 60, and 65°C incubations (Supplementary Table S3). The tSRB consortium had high fractions of the thermophiles *Thermus* (25%), *Anoxybacillus* (8.8%) and *Desulfotomaculum* (7.6%). However, the 55, 60, and 65°C incubations were dominated by the genus *Desulfotomaculum*, which was present at 98.8, 97.2, and 95.5% respectively (Supplementary Table S4).

### Effect of Addition of Nitrite or Nitrate to tSRB Enrichments in Batch Culture

**FIGURE 6:**
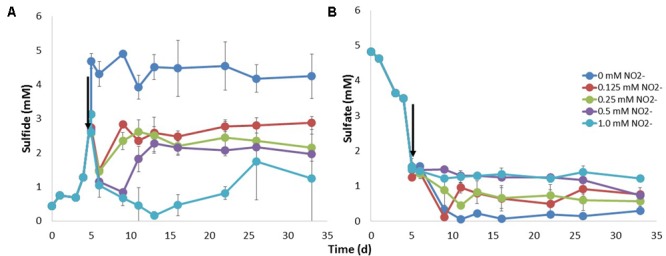
Inhibition of sulfate reduction by tSRB with nitrite. A tSRB consortium enriched at 60°C (**Figure 5**) was grown at this same temperature in medium with 20 mM lactate and 10 mM sulfate. The concentrations of **(A)** sulfide and **(B)** sulfate are shown as a function of time. Nitrite was injected at midlog phase (↓) in concentrations as indicated.

The effect of nitrite on sulfate reduction was tested at 60°C, using the 60°C tSRB enrichment of **Figure 5**, which contained mostly *Desulfotomaculum* (Supplementary Table S4). Addition of nitrite at mid-log phase caused an instant drop in sulfide concentration (**Figure 6A**), due to chemical reaction of nitrite with sulfide. Addition of the lowest concentrations of 0.125 and 0.25 mM nitrite inhibited sulfide production only transiently. However, the final concentration of sulfide produced remained below that of the untreated culture (**Figure 6A**). Sulfate concentrations remained constant following addition of 0.5 or 1 mM nitrite, while some sulfate reduction was observed at lower nitrite concentrations (**Figure 6B**).

**FIGURE 7:**
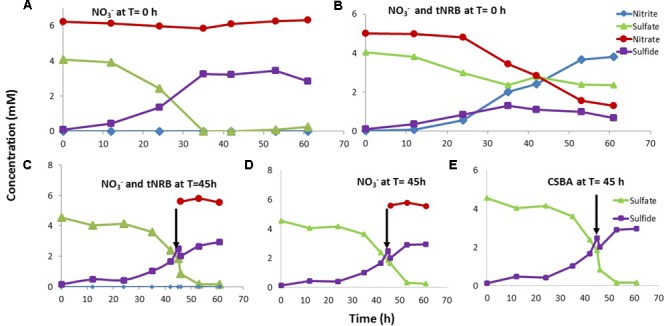
Effect of addition of nitrate or of nitrate and tNRB on sulfate reduction by tSRB consortia grown at 60°C. Nitrate **(A)** or nitrate and tNRB **(B)** were added at T = 0 h **(A,B)** or at midlog phase of sulfate reduction at T = 45 h **(C,D)**; 100 μl of CSBA medium was added as a control at T = 45 h **(E)**. Data are averages for duplicate incubations.

When nitrate was added it was not reduced and reduction of sulfate to sulfide was complete in 35 h (**Figure 7A**). However, when both nitrate and tNRB (a mixture of *Geobacillus* sp. strains TK004 and TK005) were added at time zero reduction of nitrate to nitrite was observed from 24 h onward. This inhibited the reduction of sulfate, which remained constant from 35 h onward. Sulfide concentrations decreased from 35 h onward (**Figure 7B**); development of a yellow color indicated the formation of polysulfides from reaction of nitrite and sulfide. The addition of nitrate and actively growing tNRB or of nitrate only to a tSRB culture at mid-log phase did not give nitrate reduction and no inhibition of sulfate reduction was observed (**Figures 7C,D**). This was because the time necessary for tNRB to grow (**Figure 7B**: 24 h), exceeded the time needed for the tSRB culture to grow to completion (**Figures 7C–E**: 5 h). Overall the results indicated that addition of tNRB and nitrate at time zero resulted in inhibition of tSRB activity due to formation of nitrite. Inhibition was not observed when only nitrate was added or when tNRB and nitrate were added at midlog phase.

### Addition of Nitrate, Nitrate and tNRB or Nitrite to Continuous Cultures of tSRB

**FIGURE 8:**
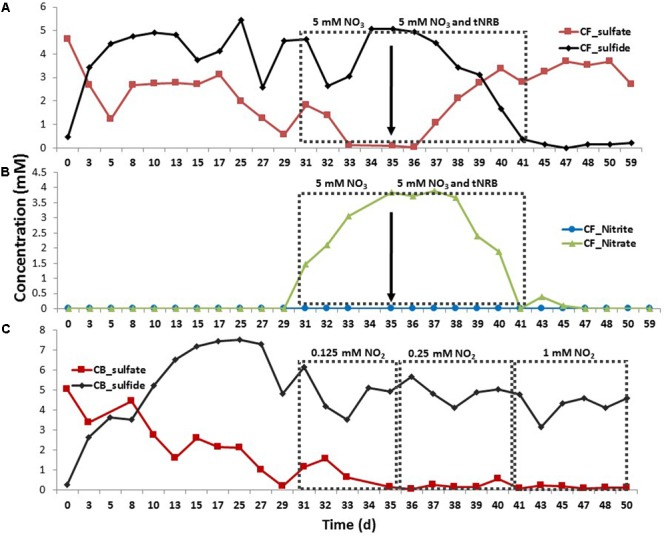
Effect of addition of nitrate, of nitrate and tNRB, or of nitrite on tSRB activity during continuous culture conditions at 60°C. The concentrations (mM) are shown as a function of time (days) for **(A)** sulfate and sulfide in chemostat F (CF), **(B)** nitrate and nitrite in CF and **(C)** sulfate and sulfide in chemostat B (CB). CF was injected with medium containing lactate and sulfate. This was switched to medium with lactate, sulfate and nitrate as indicated; tNRB were added as indicated (↓). CB was injected with medium containing lactate and sulfate with addition of 0.125, 0.250, and 1.0 mM of nitrite as indicated. Note that the time scale is not linear.

A continuous culture (chemostat) of tSRB, inoculated with the 60°C enrichment of **Figure 5**, was fed with CSBA medium with lactate and 5 mM sulfate at a dilution rate of 0.33 day^-1^; 5 mM nitrate was included in the inflowing medium from day 31 to day 41. This led to a gradual increase in the nitrate concentration in the chemostat to 4 mM from day 31–35 (**Figure 8B**). This indicated that nitrate was not reduced. Indeed, the reduction of sulfate to sulfide was not affected (**Figure 8A**: day 31–35). However, when a single dose of tNRB was added to the chemostat on day 36 (**Figures 8A,B**: ↓), the nitrate concentration decreased from 4 mM to zero from day 36 to day 41 (**Figure 8B**). The concentration of sulfide decreased from 5 to 0.2 mM, whereas the concentration of sulfate increased from 0 to 3 mM (**Figure 8A**). The development of turbidity and yellow color indicated formation of sulfur and polysulfide (S-PS), respectively. Nitrite was not detected, likely because it reacted with sulfide to form S-PS. When the medium was switched on day 41 to medium with sulfate only (no nitrate), the tSRB did not recover as indicated by zero sulfide and 3.7 mM sulfate from day 41–59 (**Figure 8A**). Addition of 0.125, 0.25, or 1 mM nitrite to the inflowing medium in another similarly run chemostat did not inhibit the reduction of sulfate of which the concentration remained at zero (**Figure 8C**). However, sulfide concentrations dropped. Nitrite was again not detected indicating reaction of nitrite and sulfide (**Figure 8C**). Note that the average concentration of added nitrite over the indicated time periods would be approximately half of that added to the inflowing medium (0.062, 0.125, and 0.5 mM). The gradual addition of nitrite appeared to affect tSRB in continuous culture less than the addition of a single dose in batch culture (**Figures 6A, 8C**).

## Discussion

Oil production from Terra Nova started in 2002 with seawater injection being required soon after (Haugen et al., 2007). Souring became evident after about 8 years of seawater flooding (Sharpe et al., 2015) and nitrate and nitrite were injected as a souring control strategy from 2014 onward. Because of the high reservoir temperature of 95°C some regions will be abiotic and microbial growth is expected only in regions where the temperature is below 80°C (**Figure 1**). It is generally thought that most souring occurs in the NIWR, where sulfate-containing IW comes in contact with oil. Because injected sea water has a high sulfate concentration (**Table 1**) both mesophilic SRB and thermophilic SRM may contribute to souring (Thrasher and Vance, 2005; Torsvik and Sunde, 2005). The produced sulfide then travels through the reservoir. Its appearance in produced water can take a long time (i.e., 8 years in the case of Terra Nova) due to sulfide scavenging by reservoir rock (Thrasher and Vance, 2005).

Uncovering the microbiology of the NIWR is thus relevant, but is challenging because only samples of IW and of produced water are available in most cases. Reversing the flow of IW would allow a more direct collection of samples from the NIWR, but such samples are only rarely available (Bødtker et al., 2009). Microbial community data obtained for produced water samples cannot be pinpointed to a particular region. These samples may contain IW microbes, which passed through the reservoir and were heat killed along the way, as well as sessile bacteria growing on the walls of pipelines transporting produced water and oil to the FPSO (**Figure 1**). Low microbial counts and activities are commonly observed in produced waters from high temperature reservoirs (Bonch-Osmolovskaya et al., 2003; Birkeland, 2005; Kaster et al., 2007). Likewise, the seawater used as IW may contain thermophiles from the reservoir or pipeline walls, due to the continuous discharge of de-oiled, high temperature produced water (**Figure 1**). Therefore, a variety of approaches including community analysis, culturing and determination of temperature optima are needed to reconstruct the positioning of microbes and their activities in the NIWR.

Of the thermophiles detected in this study, *Thermoanaerobacter* spp. are fermentative bacteria, which have been frequently found in produced water samples from high temperature oil fields (Orphan et al., 2000; Pavlova-Kostryukova et al., 2014; Yeung et al., 2015). Other detected thermophiles were the fermentative bacterial taxon *Thermosipho* (Haridon et al., 2001; Dahle et al., 2008), the archaeal taxon *Thermococcus*, which is also a sulfur reducer (Zhang Y. et al., 2012; Liang et al., 2014; Lin et al., 2014; Gorlas et al., 2015) and the methanogen *Methanothermococcus*. These results indicate that regions in the NIWR and pipeline walls with the right temperature regime will harbor diverse metabolically active thermophilic and hyperthermophilic anaerobes (Slobodkin et al., 1999; Orphan et al., 2003) of which only a few have been cultured (Orphan et al., 2000). Gittel et al. (2009) reported that an enrichment of a sample, which had 56% *Archaeoglobus* sequences, had no sulfate-reducing activity. These authors were also unable to enrich tNRB from produced water samples from the Ekofisk oilfield. *Desulfotomaculum* spp. are the most documented culturable tSRB isolated from oil field environments (Nilsen et al., 1996a,b; Nazina et al., 2006; Aüllo et al., 2013), but are not often detected with next generation sequencing approaches (Müller et al., 2014; Wunderlin et al., 2014). *Thermodesulforhabdus norvegicus* and *Archaeoglobus fulgidus* strains were among the dominant tSRM detected by Nilsen et al. (1996a) in North Sea oil reservoirs and Beeder et al. (1995) isolated a novel acetate-oxidizing *T. norvegicus*, which grew at temperature of up to 74°C. Li et al. (2007) detected *Thermodesulfovibrio*, a Gram-negative tSRB, as a major component of a 16S rRNA gene clone library from a North Sea field.

Fida et al. (2016) reported the isolation of the tNRB *Petrobacter* sp. TK002 (optimum growth at 50°C) and *Geobacillus* sp. TK003 (optimum growth at 65°C), which reduced nitrate to nitrite. Although some tNRB have been reported to reduce nitrate to N_2_ or ammonium (Greene et al., 1997; Miranda-Tello et al., 2003), reduction of nitrate to nitrite appeared to be the norm for oil field tNRB (Fida et al., 2016). The addition of nitrate often does not inhibit tSRB consortia, because these lack tNRB. Such tSRB consortia are only inhibited by nitrite or by nitrate, if tNRB are also injected (**Figures 6B, 7A,B**). The inhibition of the tSRB consortia used in this study and in other work by low concentrations of nitrite (Kaster et al., 2007) indicates absence of Nrf nitrite reductase, which reduces nitrite to ammonium. Nrf protects many mesophilic SRB from inhibition by nitrite (Greene et al., 2003; Haveman et al., 2004).

**FIGURE 9:**
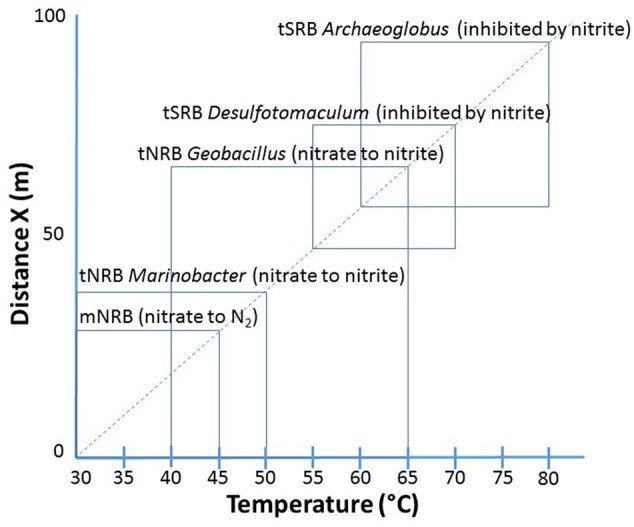
Distribution of NRB and SRB from Terra Nova identified or cultivated in this study in the NIWR. These included mesophilic NRB, tNRB *Marinobacter*, tNRB *Geobacillus*, tSRB *Desulfotomaculum* and the thermophilic sulfate-reducing archaeon (tSRA) *Archaeoglobus*. The latter was not cultivated and its range of activity was inferred from the literature. The scale on the *Y* axis is an estimate of the size of the NIWR (from 0 to 100 m) and the approximate position of the taxa in this region

Although this would suggest that injection of nitrite, which directly inhibits SRM, is preferable to the injection of nitrate this may not apply at Terra Nova, where both mesophilic and tNRB are present. Mesophilic NRB grow closest to the injection wellbore at 30–45°C (**Figure 9**). These reduce nitrate to N_2_ (**Figures 3A,B**), but have not been phylogenetically characterized. These are followed by moderately tNRB of the genus *Marinobacter* growing at 30–50°C (**Figures 3C,G-I, 9**) and these are succeeded by tNRB of the genus *Geobacillus*, which grow from 40 to 65°C (**Figure 4**; Fida et al., 2016). There is no tNRB activity at 70°C or higher temperature. The activity of tSRB of the genus *Desulfotomaculum* extends from 55 to 70°C (**Figure 5** and Supplementary Figure S3). The observation of significant fractions of *Archaeoglobus* in some samples (Supplementary Table S1: entry #28) indicates that the temperature limit for sulfate reduction may be at even higher temperature (Beeder et al., 1994; Gittel et al., 2009). Because mesophilic SRB were not detected with cultivation or 16S rRNA gene sequencing, we assume that these were largely absent from Terra Nova, where nitrate injection may displace SRB from low temperature zones. Thus at Terra Nova sulfide may be mainly produced by tSRB living in a high temperature zone (65–80°C) from which tNRB are excluded. Preventing the temperature of the NIWR to drop below 50°C would increase the production of nitrite from injected nitrate in tNRB inhabited zones (50–65°C). Transfer of this produced nitrite into the adjacent higher temperature zone (65–80°C) will inhibit the resident tSRB, if the rate of transfer exceeds the rate of sulfide production by tSRB. These conditions were apparently not met in our continuous culture study (**Figure 8C**). Injection of hot recycled produced water to keep the temperature above 50°C will increase the transfer of nitrite into tSRB-inhabited zones and is therefore a promising strategy to control souring in high temperature oil reservoirs, as suggested previously (Fida et al., 2016).

## Author Contributions

GO, CC, and TF designed and carried out the study, collected data, performed the analysis. GO wrote the manuscript. GV contributed to data interpretation and preparation of the manuscript.

## Conflict of Interest Statement

The authors declare that the research was conducted in the absence of any commercial or financial relationships that could be construed as a potential conflict of interest.

## Footnotes

1 http://ebg.ucalgary.ca/metaamp
