# Supplementary material for: Effect of Thermophilic Nitrate Reduction on Sulfide Production in High Temperature Oil Reservoir Samples
Source: Front Microbiol. 2017 Aug 29;8:1573. doi: 10.3389/fmicb.2017.01573 (PMC5581841; doi:10.3389/fmicb.2017.01573)
Supplement: Supplementary file 1 [file Data_Sheet_1.pdf]

Supplementary Material

Effect of thermophilic nitrate-reduction on sulfide production in high temperature oil reservoir samples

Gloria Ngozi Okpala[1], Chuan Chen[2], Tekle Fida[1] and Gerrit Voordouw[1*]

*** Correspondence:**
Corresponding Author: Gerrit Voordouw: voordouw@ucalgary.ca

1 Supplementary Figures and Tables

Table S1 | Microbial community compositions of samples PW1_14 (PW) and IW1_14 (IW) derived from pyrosequencing. The fractions of total reads are indicated for each taxon. Fractions in excess of 1% are indicated in bold.

#	Taxonomy	PW	IW
		V38_1843	V38_1844
1	Proteobacteria;Alphaproteobacteria;NA__DB1-14;	0	**2.1**
2	Proteobacteria;Alphaproteobacteria;NA__OCS116-clade;	0	**4.23**
3	Proteobacteria;Alphaproteobacteria;Rhodobacterales;Rhodobacteraceae;	0	0.93
4	Proteobacteria;Alphaproteobacteria;Rhodobacterales;Rhodobacteraceae;_Roseovarius;	0	0.66
5	Proteobacteria;Alphaproteobacteria;Rhodobacterales;Rhodobacteraceae;Celeribacter;	0	**5.1**
6	Proteobacteria;Alphaproteobacteria;Rhodospirillales;Rhodospirillaceae;Nisaea;	0	0.95
7	Proteobacteria;Alphaproteobacteria;Rhodospirillales;Rhodospirillaceae;Thalassospira;	0	**5.72**
8	Proteobacteria;Alphaproteobacteria;Sneathiellales;Sneathiellaceae;Sneathiella;	0	0.32
9	Proteobacteria;Alphaproteobacteria;Sphingomonadales;Sphingomonadaceae;Sphingorhabdus;	0	0.34
10	Proteobacteria;AlphaproteobacteriaRhodobacterales;Rhodobacteraceae;Pseudophaeobacter;	0	0.66
11	Proteobacteria;Betaproteobacteria;Rhodocyclales;Rhodocyclaceae;	0	**24.76**
12	Proteobacteria;Deltaproteobacteria;Desulfovibrionales;Desulfohalobiaceae;Desulfonauticus;	0.32	0
13	Proteobacteria;Gammaproteobacteria;Alteromonadales;Alteromonadaceae;Alteromonas;	0	0.35
14	Proteobacteria;Gammaproteobacteria;Alteromonadales;Alteromonadaceae;Marinobacter;	0	0.54
15	Proteobacteria;Gammaproteobacteria;Alteromonadales;Shewanellaceae;Shewanella;	0	0.96
16	Proteobacteria;Gammaproteobacteria;Oceanospirillales;Alcanivoracaceae;Alcanivorax;	0	**4.51**
17	Proteobacteria;Gammaproteobacteria;Oceanospirillales;Oceanospirillaceae;Litoribacillus;	0	0.93
18	Proteobacteria;Gammaproteobacteria;Oceanospirillales;Oceanospirillaceae;Neptuniibacter;	0	**33.2**
19	Proteobacteria;Gammaproteobacteria;Pseudomonadales;Pseudomonadaceae;Pseudomonas;	**6.88**	0.48
20	Proteobacteria;Gammaproteobacteria;Thiotrichales;Piscirickettsiaceae;Cycloclasticus;	0	**3.03**
21	Proteobacteria;Gammaproteobacteria;Thiotrichales;Piscirickettsiaceae;Methylophaga;	0	**5.53**
22	ProteobacteriaGammaproteobacteria;Alteromonadales;olwelliaceae;Colwellia;	0	0.98
23	Bacteroidetes;Flavobacteriia;Flavobacteriales;Flavobacteriaceae;Mesoflavibacter;	0	0.58
24	Bacteroidetes;Flavobacteriia;Flavobacteriales;Flavobacteriaceae;Ulvibacter;	0	**2.8**
25	Firmicutes;Clostridia;Clostridiales;Clostridiaceae-4;Caminicella;	0.2	0
26	Firmicutes;Clostridia;Thermoanaerobacterales;Thermoanaerobacteraceae;Thermoanaerobacter;	**78.2**	0
27	Thermotogae;Thermotogae;Thermotogales;Thermotogaceae;Thermosipho;	0.74	0
28	Euryarchaeota;Archaeoglobi;Archaeoglobales;Archaeoglobaceae;Archaeoglobus;	**1.7**	0
29	Euryarchaeota;Methanococci;Methanococcales;Methanococcaceae;Methanothermococcus;	**7.08**	0
30	Euryarchaeota;Methanomicrobia;Methanosarcinales;Methermicoccaceae;Methermicoccus;	**2.21**	0.01
31	Euryarchaeota;Thermococci;Thermococcales;Thermococcaceae;Thermococcus;	**2.19**	0
	**Total number of reads**	**6472**	**6857**

Table S2 | Microbial community composition of injection water and produced water samples collected in 2015 derived from Illumina sequence data. The fractions of total reads are indicated for each taxon. Fractions in excess of 1% are indicated in bold.

	Sample name	IW1_15	PW1_15	IW5_15	PW_F2_5_15
	Group name	V48_2558	V45_2319	V48_2565	V48_2566
	Number of high quality reads	50990	28927	24127	13410
#	Class/Genus				
1	*Alphaproteobacteria; Roseovarius*	**6.3**	0.9	0.4	0.0
2	*Alphaproteobacteria;Caulobacter*	0.0	0.0	0.0	**10.3**
3	*Alphaproteobacteria;Maritimibacter*	0.6	0.4	**26.5**	0.0
4	*Alphaproteobacteria;Sulfitobacter*	**22.3**	**24.5**	**2.2**	0.0
5	*Alphaproteobacteria;Thalassospira*	0.0	0.0	**1.5**	0.0
6	*Betaproteobacteria;Aquabacterium*	0.0	0.0	0.0	**13.8**
7	*Betaproteobacteria;Burkholderia*	0.0	0.0	0.0	**12.1**
8	*Betaproteobacteria;Roseateles*	0.0	0.0	0.0	**7.9**
9	*Gammaproteobacteria;Acinetobacter*	0.0	0.0	0.0	**2.9**
10	*Gammaproteobacteria;Alteromonas*	**30.6**	**18.2**	0.6	0.0
11	*Gammaproteobacteria;Beggiatoa*	0.0	0.0	0.0	**3.6**
12	*Gammaproteobacteria;Colwellia*	0.9	0.2	0.4	0.0
13	*Gammaproteobacteria;Klebsiella*	0.0	0.0	**4.7**	**16.0**
14	*Gammaproteobacteria;Marinobacter*	0.2	**2.7**	0.8	0.0
15	*Gammaproteobacteria;Methylophaga*	0.0	0.2	**11.9**	0.0
16	*Gammaproteobacteria;Neptuniibacter*	**9.2**	0.2	0.5	0.0
17	*Gammaproteobacteria;Oceanobacter*	**3.3**	**37.9**	**1.3**	0.0
18	*Gammaproteobacteria;Porticoccus*	**1.2**	0.0	**2.8**	0.0
19	*Gammaproteobacteria;Pseudomonas*	0.0	**7.1**	0.0	**26.6**
20	*Gammaproteobacteria;Shewanella;*	0.0	0.1	0.8	0.0
21	*GammaproteobacteriaThiomicrospira*	0.0	0.0	**42.0**	0.0
22	*Actinobacteria;Microbacteriaceae*	0.0	0.0	0.0	**2.9**
23	*Actinobacteria;Rhodococcus*	0.0	0.0	0.0	**2.7**
24	*Bacilli;Streptococcus*	0.0	0.0	0.0	**1.1**
25	*Bacteroidetes;Flavobacteriales*	**1.1**	0.2	0.0	0.0
26	*Bacteroidetes;Maribacter*	**3.2**	**1.3**	0.0	0.0
27	*Bacteroidetes;Polaribacter*	0.6	0.5	0.0	0.0
28	*Bacteroidetes;Psychroserpens*	**20.3**	**4.5**	0.0	0.0
29	*Flavobacteriia;Lutibacter*	0.0	0.0	**3.4**	0.0
30	*Methanococci;Methanothermococcus*	0.0	0.7	0.0	0.0
31	*Thermococci;Thermococcus*	0.0	0.4	0.0	0.0

Table S3 | Number of quality controlled (QC) reads, number of derived operational taxonomic units (OTUs) as extrapolated by Chao and Shannon diversity index for the tSRB inoculum and derived enrichments as in Figure 5.

Sample	Raw reads	QC_reads	OTUs_chao	Shannon
tSRB_inoculum	83330	29245	292	3.30
55°C	83364	26896	29	0.11
60°C	68412	19974	44	0.23
65°C	65892	17579	39	0.32

Table S4 | Microbial community compositions of tSRB inoculum and of tSRB grown at 55, 60 and 65 °C, as in Figure 5. Fractions in excess of 1% are in bold.

#Taxon (Phylum, class, genus)	tSRB_inoculum	55°C	60°C	65°C
Proteobacteria; Alphaproteobacteria; Bradyrhizobiaceae	0	0.04	0.01	0.05
Proteobacteria; Alphaproteobacteria; Caulobacter	0.08	0.23	0.34	0.43
Proteobacteria; Betaproteobacteria; Aquabacterium	0.12	0.13	0.32	0.75
Proteobacteria; Betaproteobacteria; Burkholderia	0.07	0.11	0.21	0.39
Proteobacteria; Betaproteobacteria; Roseateles	0.05	0.07	0.06	0.4
Proteobacteria; Betaproteobacteria; Thauera	**14.92**	0.01	0.01	0.25
Proteobacteria; Deltaproteobacteria; Desulfovibrio	**3.03**	0	0	0
Proteobacteria; Gammaproteobacteria; Acidibacter	0.01	0.02	0.07	0.12
Proteobacteria; Gammaproteobacteria; Acinetobacter	0.12	0.02	0.05	0.13
Proteobacteria; Gammaproteobacteria; Marinobacter	**2**	0	0	0
Proteobacteria; Gammaproteobacteria; Pseudomonas	**5.41**	0.35	0.76	**1.13**
Proteobacteria; Gammaproteobacteria; Shewanella	**1.83**	0	0	0
Actinobacteria; Microbacteriaceae	0.03	0.05	0.09	0.13
Actinobacteria; Propionibacterium	0.53	0.04	0.16	0.05
Bacteroidetes; Sphingobacteriia; Sediminibacterium	0.03	0.04	0.15	0.09
Deinococcus-Thermus; Deinococci; Thermus	**25.31**	0	0.01	0
Firmicutes; Bacilli; Anoxybacillus	**8.82**	0	0	0
Firmicutes; Bacilli; Staphylococcus	0.1	0	0.13	0.05
Firmicutes; Clostridia; Desulfitispora	**2.51**	0	0	0
Firmicutes; Clostridia; Desulfotomaculum	**7.56**	**98.76**	**97.17**	**95.47**
Euryarchaeota; Methanomicrobia; Methanoculleus	**2.7**	0	0	0

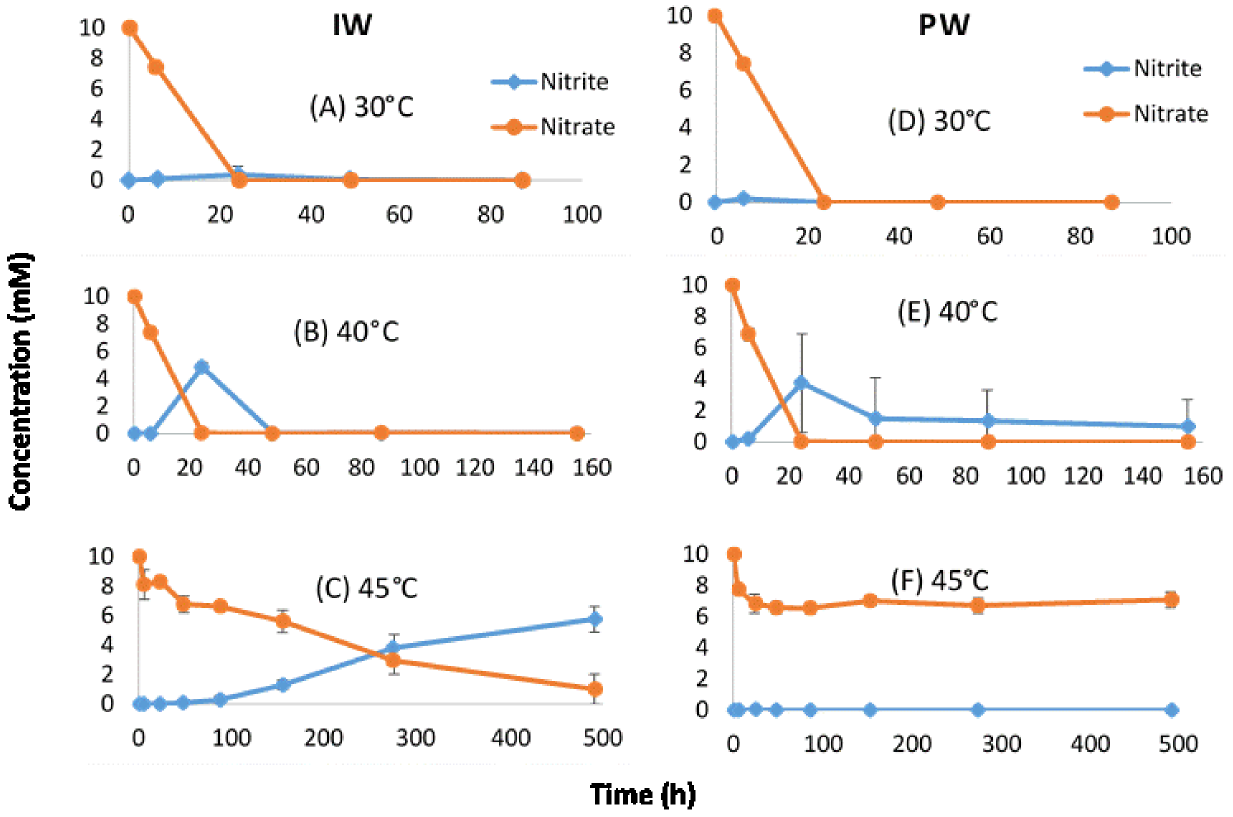

Figure S1: Temperature dependence of nitrate reduction of IW1_15 (a-c) and PW1_15 (d-f) samples, collected from the Terra Nova oil field in January 2015. The medium containing 3 mM VFA and 10 mM nitrate was inoculated with 1 ml of 50-fold concentrated IW or PW. The data are averages and standard deviations for three different incubations for each temperature.

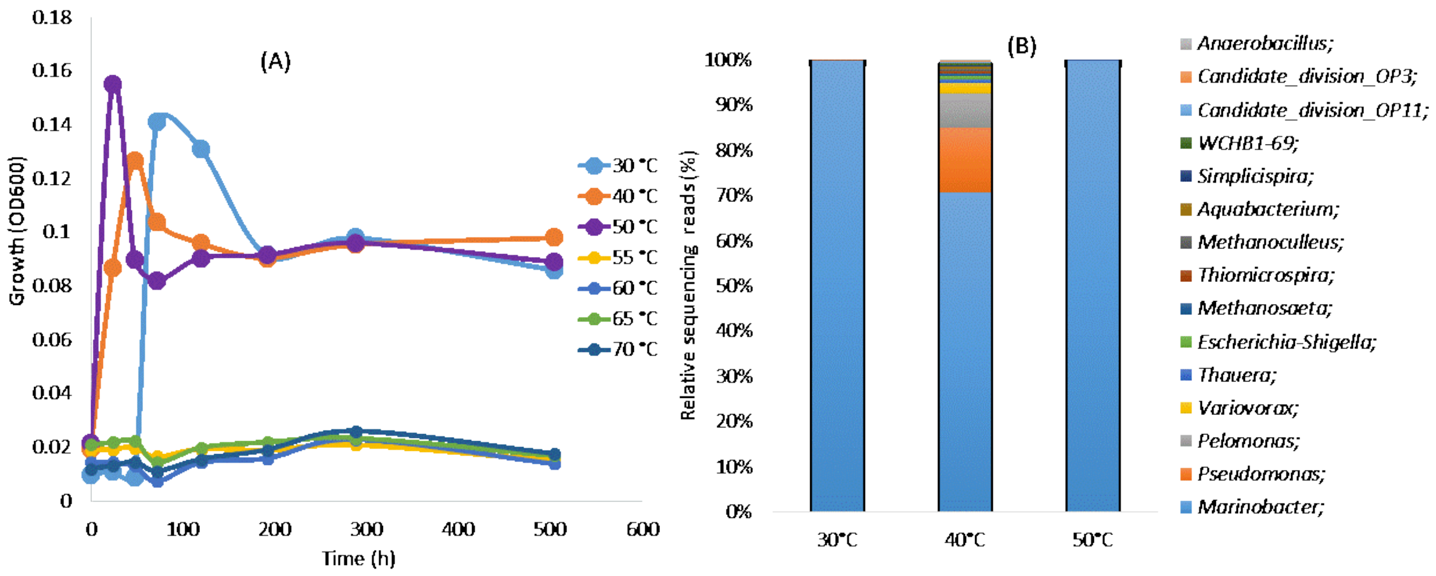

Figure S2. Effect of incubation temperature on growth and community composition of cultures inoculated with the IW5_15 primary tNRB enrichment, obtained at 50°C. Growth (A) and community composition (B) are shown for cultures at the indicated temperatures.

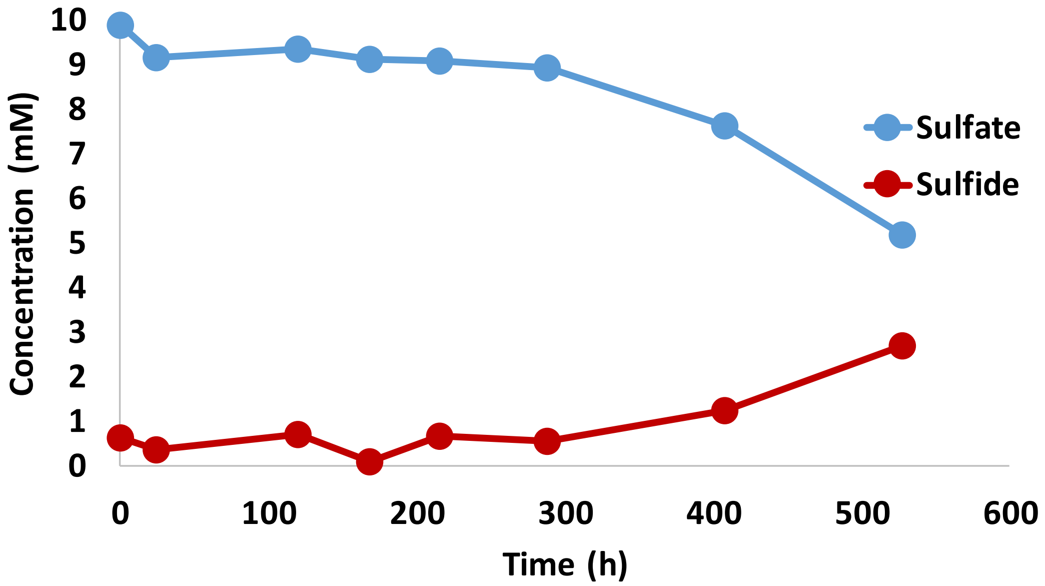

Figure S3. Sulfate reduction by tSRB enriched from PW1_14 at 70°C.
